# Monitoring Phytate Hydrolysis Using Serial Blood Sampling and Feather Myo-Inositol Levels in Broilers

**DOI:** 10.3389/fphys.2020.00736

**Published:** 2020-06-26

**Authors:** Elizabeth Greene, Barbara Mallmann, Jonathan W. Wilson, Aaron J. Cowieson, Sami Dridi

**Affiliations:** ^1^Department of Poultry Science, University of Arkansas, Fayetteville, AR, United States; ^2^DSM Animal Nutrition and Health, Kaiseraugst, Switzerland

**Keywords:** phytate hydrolysis, myoinositol, phytase, gene expression, feather, broilers

## Abstract

Phytate forms insoluble precipitates with various cations that are recalcitrant to digestion in poultry. Dietary supplementation with exogenous phytase has been shown to improve phytate solubility and digestibility and, in turn, improve animal growth performance. Although the kinetics of phytate hydrolysis by exogenous phytase are well described *in vitro*, the progression of the reaction *in vivo* is still not well defined. The aim of the present study was, therefore, to monitor the kinetic variation of myo-inositol (myo-Ins) levels in both circulation and feather following exogenous phytase supplementation. In experiment 1, 4 week-old male broilers were individually housed with *ad libitum* access to water and a standard commercial diet. Birds were maintained under environmental temperature of 24°C and 30% RH. Birds were cannulated in the cutaneous ulnar vein on the right wing and remained untouched for 3 days. On the day of the experiment, birds were randomly divided into three body weight-matched groups and fed either the control diet, the control diet-supplemented with myo-Ins or Ronozyme HiPhos (0.06%, DSM Nutritional Products, Switzerland) for 10 h. In the experiment 2, birds were fed only HiPhos for 30 h. Growing feathers and blood were collected at baseline and then every 2 h for 10 h (experiment 1) and 30 h (experiment 2) post-prandially. Plasma and feather myo-Ins levels were determined by UHPLC-MS/MS. The relative expression of inositol polyphosphate-1-phosphatase (INPP1), inositol hexakisphosphate kinase 1-3 (IP6K1-3), inositol-3-phosphate synthase (ISYNA), and multiple inositol-polyphosphate phosphatase 1 (MNPP1) genes in blood and feathers was determined by real-time qPCR using 2^–ΔΔCt^ method. Plasma and feather myo-Ins levels were significantly increased by HiPhos at 6 h to 8 h post-prandial. The mRNA abundances of INPP1, IP6K1, and ISYNA in the circulation were significantly down regulated at all periods compared to the baseline levels. IP6K2, IP6K3, and MINPP1 gene expression, however, was up regulated at 8 h post-prandial and then returned to the baseline levels. In feathers, the expression of INPP1 was induced at 8 h post-prandial and remained higher compared to the baseline. The expression of IP6K2, IP6K3, and MINPP1 was down regulated during the first 10 h and then returned to baseline levels for the rest of the post-prandial period. Taken together, our data show that phytase modulates the expression of genes associated with myo-Ins metabolism and generates release of myo-Ins in both circulation and feather at 6–10 h post-feeding. Feather myo-Ins concentration could be used as a non-invasive method to monitor phytate hydrolysis in practice.

## Introduction

In plants, a large proportion of the phosphorous (P) is bound in the form of phytate, the mixed salt of phytic acid (myo-inositol hexakisphosphate; InsP6) (Plimmer and Page, 1913; Maga, 1982; Eeckhout and De Paepe, 1994; Viveros et al., 2000). Phytate-P as a percentage of total P is highest in the cereal grains wheat and corn (Viveros et al., 2000), and it is estimated that poultry diets contain approximately 2.5–4.0 g of phytate-P per kg of feed (Ravindran, 1995). Phytase and phosphatases are the enzymes responsible for the catalysis of the stepwise hydrolysis of phytate to inorganic phosphorous and inositol (with lower-phosphorylated inositol intermediates). Endogenous activity of these enzymes within the digestive tract of poultry is concentrated in the small intestine where the relatively high pH and presence of various cations renders phytate solubility low, compromising phytate-P retention (Ravindran, 1995; Maenz and Classen, 1998; Rutherfurd et al., 2004; Cowieson et al., 2006). This, significantly impairs P retention and in turn creates complex environmental pressure (Waldroup, 1999). Additionally, phytate has anti-nutritional effects, as it can interact with multiple minerals and proteins, particularly calcium, iron, and zinc, and subsequently impact absorption of these compounds (Cheryan and Rackis, 1980). Consequently, the majority of commercial poultry diets have been supplemented with exogenous phytase (Bedford, 2000) to not only improve the use and absorption of phytate-P, but to also decrease the anti-nutritive effects of this compound (Mullaney et al., 2000; Selle and Ravindran, 2007). This supplementation has beneficial effects on broiler performance, including increased body weight gain, feed efficiency (Dilger et al., 2004; Dos Santos et al., 2013), and nutrient utilization (Liu et al., 2014). Interestingly, the effects of dietary phytase can be greater than expected from increased nutrient release alone (Coweison et al., 2015). These effects may be due, in part, to the production/release of myo-inositol (myo-Ins), as it has been shown to have effects on mammalian metabolism, as it can act as an insulin memetic (Chhetri, 2019; Gonzalez-Uarquin et al., 2019). In poultry, however, the kinetics of phytate hydrolysis by exogenous phytase is still not well defined. Furthermore, information on myo-Ins absorption, transport within the body, and metabolism in poultry is sparse. Therefore, the objective of the present study was to monitor the kinetic variation of circulating myo-Ins levels following exogenous phytase supplementation. As myo-Ins has been shown to be involved in hirsutism treatment (Minozzi et al., 2008), and as feathers have been demonstrated to be a new biomonitoring tool (Jaspers et al., 2011), we extended our analysis to growing feathers.

## Materials and Methods

### Care and Use of Animals

This study was conducted in accordance with the National Institutes of Health recommendations guide for laboratory animal use and care. All animal care and procedures were approved by the Institutional Animal Care and Use Committee at the University of Arkansas (protocol # 16084).

### Animal Procedure and Environment

**FIGURE 1:**
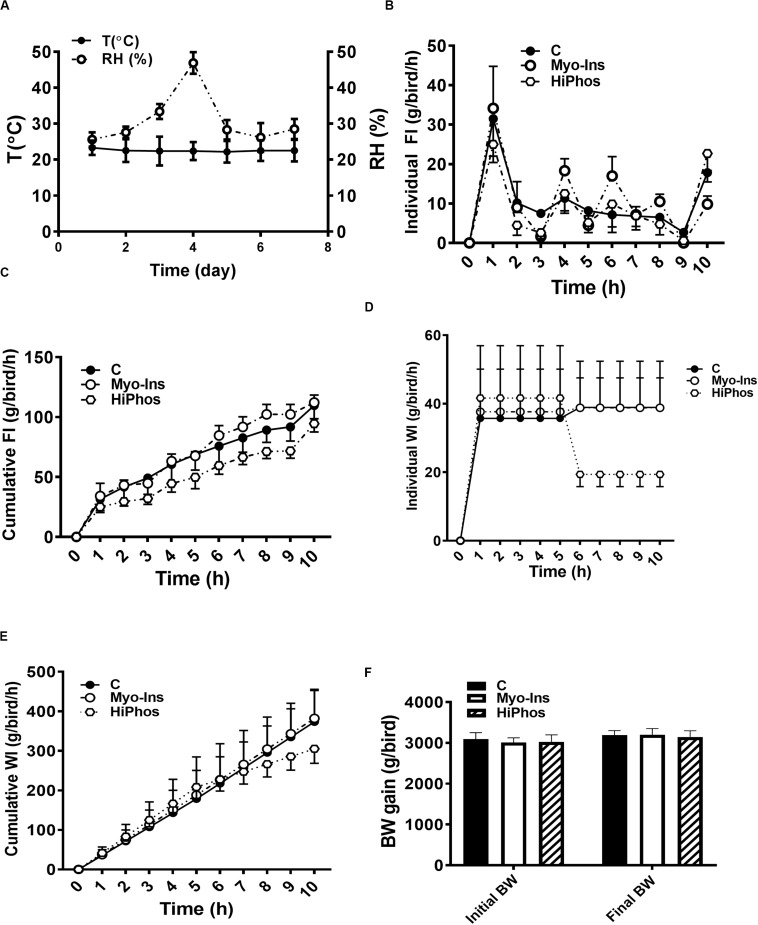
Effect of myo-Ins and HiPhos supplementation during experiment 1 on chicken feed intake, water intake, and body weight. **(A)** Environmental condition (RH and T°) of the chambers. Myo-Ins and HiPhos did not affect individual and cumulative feed intake **(B,C)**. HiPhos decreased individual **(D)** and cumulative **(E)** water intake. There was no change in BW **(F)**. Data are presented as mean ± SEM. BW, body weight; Myo-Ins, myo-inositol.

Two experiments were conducted. In the first experiment, 4 week-old male broilers (Cobb 500, *n* = 36) were transferred to individual cages and acclimatized for 1 week to the laboratory housing conditions with *ad libitum* access to water and a diet that meets the usual integration energy and nutrient allowances (Table 1). Birds were maintained under environmental temperature of 24°C and 20% RH (Figure 1A). Birds were cannulated in the cutaneous ulnar vein on the right wing, using the technique of Bayer et al. (2012). Briefly, feathers were removed, and the area surrounding the vein was cleaned with 70% ethanol. Lidocaine cream (AneCream, Focus Health Group, Knoxville, TN) was topically applied to the cannulation site, followed by a subcutaneous injection of 2% lidocaine HCl to mitigate pain. A 24G, $\frac{3}{4}$ inch winged catheter (B. Braun, Germany) was inserted into the vein, patency confirmed, and fixed in place by sutures (Ethilon nylon monofilament 4-0, Ethicon, Somerville, NJ) on both sides of the cannula. A 10% heparin solution was used to maintain patency and prevent clot formation. On the day of the experiment, birds were randomly divided into three body weight-matched groups and fed either the control diet (C, Table 1) or diets supplemented with *myo-Ins* (Sigma-Aldrich, St. Louis, MO) at 0.30% (Myo-Ins) or Ronozyme HiPhos (0.06%, DSM Switzerland) (HiPhos). Feed and water intake were recorded hourly, and body weights were recorded at the beginning and end of the experiment (10 h). The second experiment was similar to the first one, except that two diets were used (C at the baseline vs. HiPhos) for a duration of 30 h.

**TABLE 1:** Ingredient and nutrient composition of the basal experimental diet.

Ingredient, %	Starter	Grower
Corn	61.720	66.690
Soy bean meal, 46%	33.112	28.016
Poultry fat	1.899	2.248
Dicalcium phosphate	0.792	0.663
Limestone	1.130	1.096
Salt	0.282	0.285
Sodium bicarbonate	0.120	0.120
DL-methionine	0.328	0.283
L-lysine HCl	0.248	0.237
L-threonine	0.102	0.096
Choline chloride, 60%	0.028	0.026
Vitamin premix^1^	0.100	0.100
Trace mineral premix^2^	0.100	0.100
Selenium premix^3^	0.020	0.020
Santoquin^4^	0.020	0.020
**Calculated nutrients, %**		
Dry matter	87.94	87.81
AMEn, kcal/kg	3035	3108
Crude protein	21.20	19.10
AID Lys	1.18	1.05
AID Met	0.61	0.54
AID TSAA	0.89	0.80
AID Thr	0.77	0.69
AID Trp	0.22	0.19
AID Arg	1.27	1.12
AID Ile	0.79	0.70
AID Val	0.86	0.78
Total calcium	0.74	0.68
Total phosphorus	0.56	0.51
Available phosphorus	0.30	0.27
Sodium	0.17	0.17
Potassium	0.88	0.80
Chloride	0.25	0.25
Magnesium	0.17	0.16
Copper	16.86	16.22
Selenium	0.20	0.20
Choline	1,750	1,650
Linoleic acid	1.20	1.30
**Analyzed nutrients, %**		
Crude protein	21.00	18.65

*^1^Supplied per kilogram of diet: manganese, 100 mg; magnesium, 27 mg; zinc, 100 mg; iron, 50 mg; copper, 10 mg; iodine, 1 mg. ^2^Supplied per kilogram of diet: vitamin A, 30,863 IU; vitamin D_3_, 22,045 ICU; vitamin E, 220 IU; vitamin B_12_, 0.05 mg; menadione, 6.0 mg; riboflavin, 26 mg; d-pantothenic acid, 40 mg; thiamine, 6.2 mg; niacin, 154 mg; pyridoxine, 11 mg; folic acid, 3.5 mg; biotin, 0.33 mg. ^3^Supplied 0.12 mg of selenium per kg of diet.*

### Sample Collection

Growing feathers and blood were collected at baseline and then every 2 h for 10 h (experiment 1) and 30 h post-prandial (experiment 2). Growing feathers were collected from the left breast tract, and the living tissues (pulp) were sectioned, rinsed in PBS, snap frozen in liquid nitrogen, and stored at −80°C until use. 250 μL of blood was collected for RNA isolation and gene expression, with the remainder placed in a heparinized tube for plasma separation. All samples were stored at −80°C until analysis.

### Circulating and Feather Myo-Inositol Measurement

After addition of internal standard (Myo-Ins and Myo-Ins-C-d6), plasma protein precipitation is performed with acetonitrile. Feather myo-Ins samples were prepared in duplicate as described by Frieler et al. (2009). After centrifugation, the supernatant is injected in LC-MS/MS system with a turbo spray ionization and analyzed by ultrahigh performance liquid chromatography 1290 Agilent system coupled with API4000 quadrupole mass spectrometer from ABsciex according to the method of Leung et al. (2011). A Poroshell 120 Hilic-phase column (100 mm × 2.1 mm i.d.; particle size, 2.7 μm) was used. The mobile phase A was a solution of water/50 mM ammonium formate/Formic acid (950/50/1; v/v/v) and the mobile phase B was a solution of acetonitrile/50 mM ammonium formate/Formic acid (950/50/1; v/v/v). The system ran under gradient mode at a flow rate of 0.6 mL/min for 2.5 min: 0 to 0.3 min held at 2.0 % of A, then 0.3 to 1.5 min linear increased to 20% of A, then decreased to 2% of A in 0.1 min and held till 2.5 min at 2% of A. The retention times for myo-Ins / myo-Ins-C-d6 were at 1.3 min. The mass spectrometer was operated in ESI negative mode. The source was set as follows: Temperature at 650°c, curtain gas at 40, transfer voltage at −4500, gas1 at 80, gas2 at 60 and CAD at 12. The multiple reaction monitoring mode transitions for the ion pairs were: Q1/Q3 = 178.8/86.6 m/z for myo-Ins and 184.9/102.0 for the internal standard. Data acquisition of extracted ion chromatograms, identification, integration and quantification are performed by Analyst^®^ software from AB Sciex. Quantification was performed by applying an external calibration. Daily performance of the system was monitored with calibration and quality control samples ran in each analytical batch.

### RNA Isolation and Quantitative Real-Time PCR

Total RNA was extracted using Trizol reagent (for feather) or Trizol LS (for blood) (Life Technologies, Carlsbad, CA) according to the manufacturer’s instructions. RNA integrity and quality was assessed using 1% agarose gel electrophoresis and RNA concentrations and purity were determined for each sample by Take 3 Micro-Volume Plate using Synergy HT multi-mode micro plate reader (BioTek, Winooski, VT). RNA samples were DNase treated, reverse transcribed using qScript cDNA Synthesis Supermix (Quanta Biosciences, Gaithersburg, MD), and amplified by real-time quantitative PCR (Applied Biosystems 7500 Real Time System) with PowerUp SYBR green master mix (Life Technologies, Carlsbad, CA, United States) as previously described (Rajaei-Sharifabadi et al., 2017). The qPCR cycling conditions were 50°C for 2 min, 95°C for 10 min followed by 40 cycles of a two-step amplification program (95°C for 15 s and 58°C for 1 min). At the end of the amplification, melt curve analysis was applied using the dissociation protocol to exclude contamination with unspecific PCR products. Relative expression of the target genes was determined using the 2^–ΔΔCt^ method, with normalization to 18 s rRNA as a housekeeping gene (Schmittgen and Livak, 2008). Oligonucleotide primer sequences specific for chicken are presented in Table 2.

**TABLE 2:** Oligonucleotide real-time qPCR primers.

Gene	Accession number^a^	Primer sequence (5′→ 3′)	Orientation	Product size (bp)
*MINPP1*	NM_204644	CGAGCACTTTGGATGCAATG	Forward	60
		CCGTCAAATCTGCGAGCTTAG	Reverse	
*IP6K1*	XM_015292893	GGACGTGGTCTTTCCATCGA	Forward	61
		TGCCGTTGTGGAGATACTGGTA	Reverse	
*IP6K2*	NM_001030596	TGTGTCCTCCGGTTTAATGACA	Forward	59
		TGGTGCTCCCGCTGGATA	Reverse	
*IP6K3*	XM_418033	GTGTTTGAACAAGACAGGAGACTCA	Forward	75
		TGCCTTCGGTTGATTTCCTATT	Reverse	
*ISYNA1*	XM_015300143	GAGCTGTGTCAGCGCATCAC	Forward	62
		CTGTGGAAGCTCTGGAATTCG	Reverse	
*INPP1*	XM_015272577	TCGGAACCAGCGCTTCTC	Forward	59
		CCTCCTGGATGAGGACATCTG	Reverse	
*18S*	AF173612	TCCCCTCCCGTTACTTGGAT	Forward	60
		GCGCTCGTCGGCATGTA	Reverse	

*^a^Accession number refer to Genbank (NCBI). INPP1, inositol polyphosphate-1-phosphatase; IP6K1-3, inositol hexakisphosphate kinase 1-3; ISYNA, inositol-3-phosphate synthase; MINPP1, multiple inositol-polyphosphate phosphatase 1.*

### Statistical Analysis

Data were analyzed by two-way repeated measures ANOVA with diet (C, Myo-Ins, and HiPhos) and time as factors. If ANOVA revealed significant effects, the means were compared by Tukey multiple range test using the Graph Pad Prism version 6.00 for Windows (Graph Pad Software, La Jolla, CA, United States), and differences were considered significant at *P* < 0.05.

## Results

During the course of the first experiment, temperature and relative humidity averaged 24°C and 30% RH, respectively (Figure 1A). Hourly individual and cumulative feed intake was highest for the myo-Ins group and lowest for the HiPhos group (Figures 1B,C). After 5 h, water intake was lower for the phytase-fed birds (Figures 1D,E). There was no significant change in body weight over the course of the experiment (Figure 1F).

**FIGURE 2:**
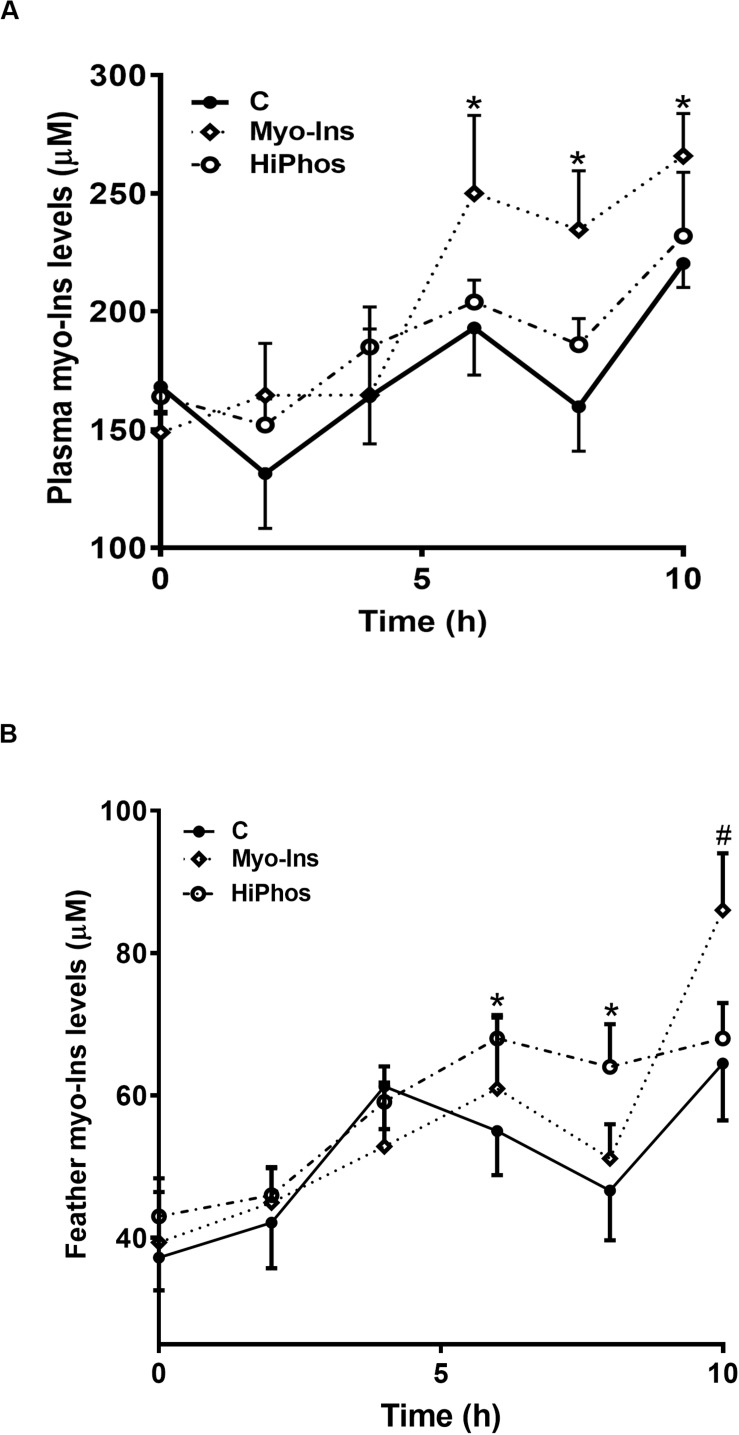
Effect of myo-Ins and HiPhos supplementation during experiment 1 on plasma and feather myo-Ins levels. Myo-Ins-enriched diets increased plasma myo-Ins concentrations 6–10 h post-prandial **(A)**. HiPhos supplementation increased feather myo-Ins concentrations 6–8 h post-prandial **(B)**. Myo-Ins supplementation increased myo-Ins concentrations in feather at 10 h post feeding. Data are presented as mean ± SEM. *Indicates significant difference compared to the control diet at *P* < 0.05. ^#^Indicates significant difference compared to the control diet at *P* < 0.01.

Plasma myo-Ins levels were increased in the myo-Ins group from 6 to 10 h (Figure 2A), whereas feather myo-Ins concentrations were increased by HiPhos after 6 h, and by myo-Ins supplementation only after 10 h (Figure 2B).

**FIGURE 3:**
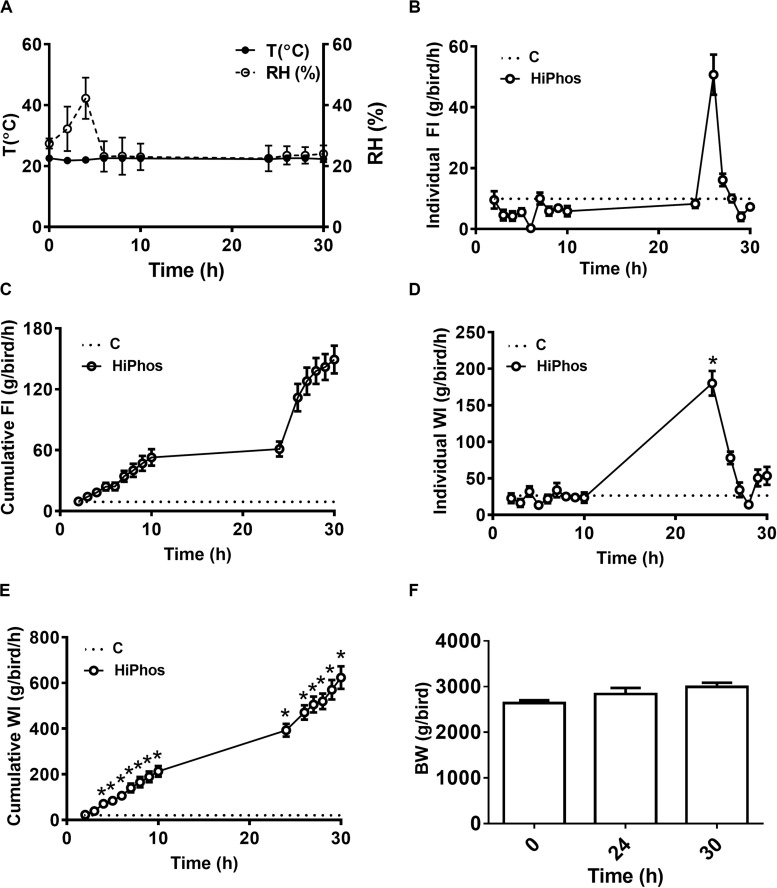
Effect of HiPhos supplementation during experiment 2 on feed intake, water intake, and body weight over 30 h in broilers. **(A)** Environmental condition (RH and T°) of the chambers. HiPhos affected individual and cumulative feed intake and individual and cumulative water intake after 24 h **(B–E)**. There was no change in BW over time **(F)**. Data are presented as mean ± SEM. *Indicates significant difference compared to the baseline (**C**, 0 h) at *P* < 0.05.

**FIGURE 4:**
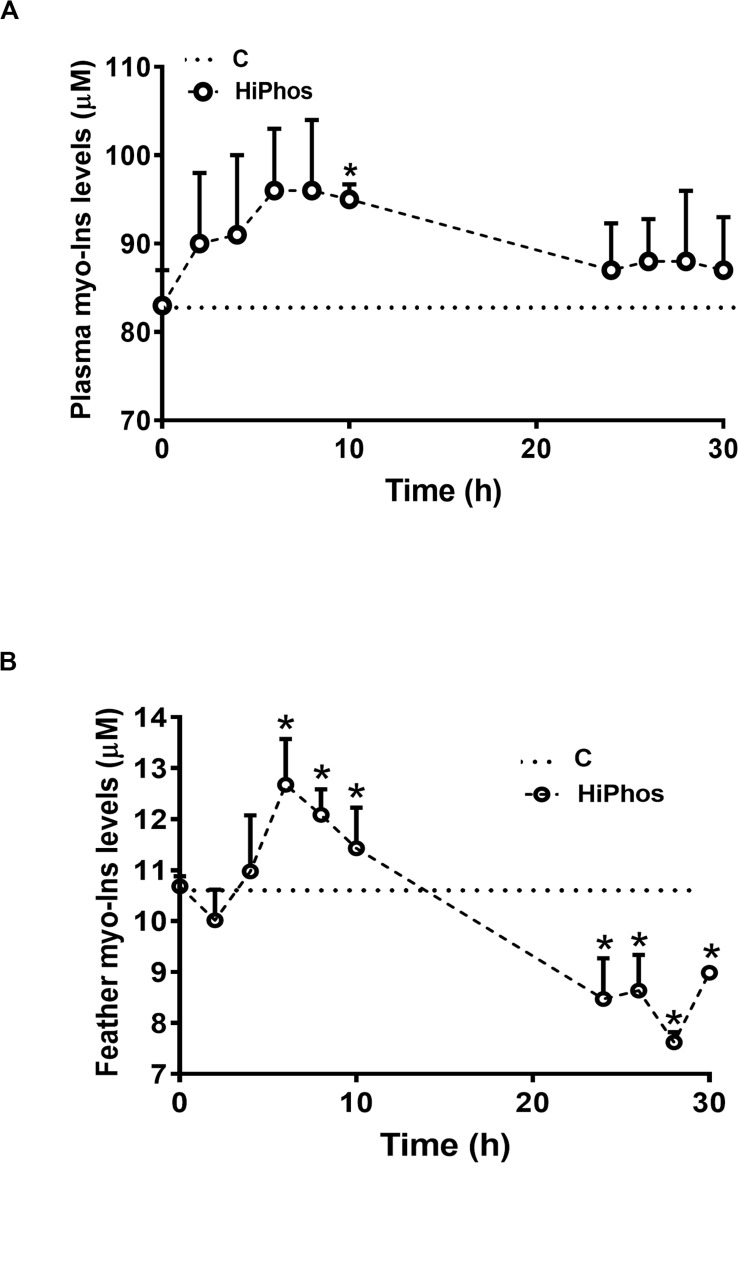
Effect of HiPhos supplementation during experiment 2 on plasma and feather myo-Ins levels over 30 h. HiPhos increased plasma **(A)** and feather **(B)** myo-Ins concentrations 6–10 h post-prandial. Plasma levels returned to baseline, whereas feather levels fell fellow baseline after 24 h. Data are presented as mean ± SEM. *Indicates significant difference compared to the baseline (C, 0 h) at *P* < 0.05.

In the second experiment, temperature and relative humidity averaged 20°C and 22%, respectively (Figure 3A). Individual feed and water remained relatively constant, with a peak in intake at 26 h compared to the beginning of the experiment (Figures 3B,D), and cumulative increases over time (Figures 3C,E). Body weight of birds did not significantly change over the course of the experiment (Figure 3F). A similar pattern was seen to the first experiment, whereby plasma (Figure 4A) and feather (Figure 4B) myo-Ins levels were increased after 6–10 h. In plasma, levels returned to baseline after 24 h, whereas in feather, myo-Ins levels fell below baseline after 24 h (Figures 4A,B).

**FIGURE 5:**
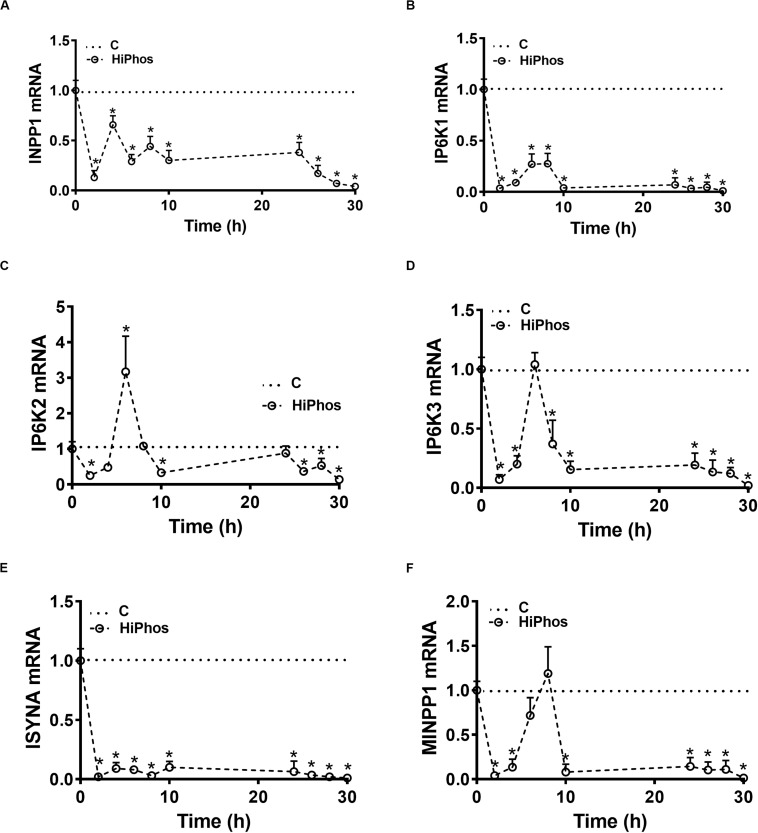
Effect of HiPhos supplementation during experiment 2 on gene expression of myo-inositol metabolism in whole blood. The expression of INPP1 **(A)**, IP6K1 **(B)**, IP6K2 **(C)**, IP6K3 **(D)**, ISYNA **(E)**, and MINPP1 **(F)** was measured using real-time qPCR and the relative expression was determined by 2^– ΔΔCt^ method (Schmittgen and Livak, 2008). Data are presented as mean ± SEM. *Indicates significant difference compared to the baseline (C, 0 h) at *P* < 0.05. INPP1, inositol polyphosphate-1-phosphatase; IP6K1-3, inositol hexakisphosphate kinase 1-3; ISYNA, inositol-3-phosphate synthase; MINPP1, multiple inositol-polyphosphate phosphatase 1.

**FIGURE 6:**
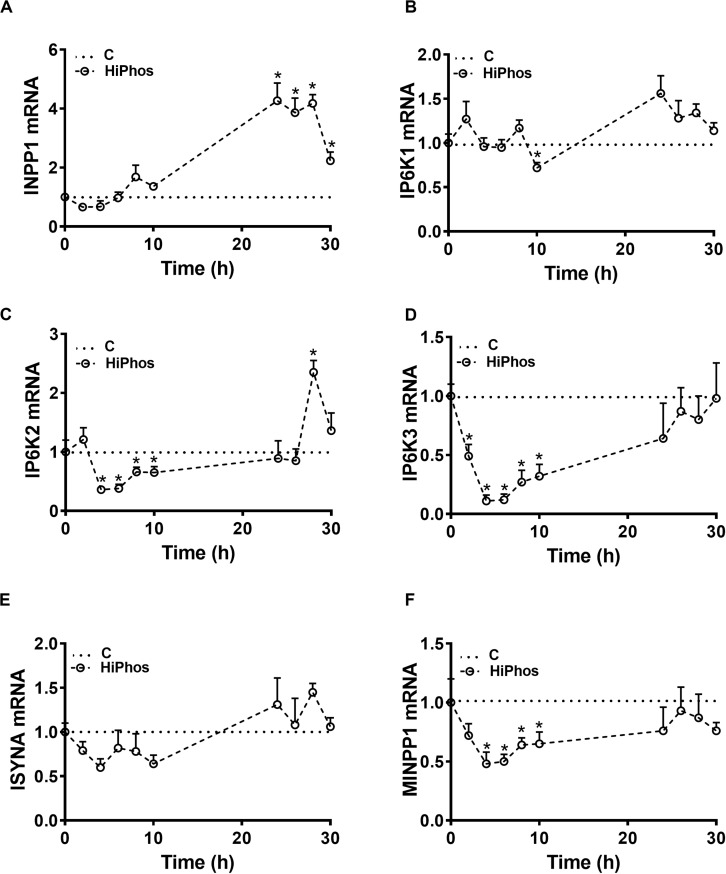
Effect of HiPhos supplementation during experiment 2 on gene expression of myo-inositol metabolism in feather. The expression of INPP1 **(A)**, IP6K1 **(B)**, IP6K2 **(C)**, IP6K3 **(D)**, ISYNA **(E)**, and MINPP1 **(F)** was measured using real-time qPCR and the relative expression was determined by 2^– ΔΔCt^ method (Schmittgen and Livak, 2008). Data are presented as mean ± SEM. *Indicates significant difference compared to the baseline (**C**, 0 h) at *P* < 0.05. INPP1, inositol polyphosphate-1-phosphatase; IP6K1-3, inositol hexakisphosphate kinase 1-3; ISYNA, inositol-3-phosphate synthase; MINPP1, multiple inositol-polyphosphate phosphatase 1.

To further investigate phytate metabolism, the gene expression of enzymes involved in phytate degradation and myo-Ins formation in circulation and feather were measured in experiment 2. In circulation, relative expression of inositol polyphosphate-1-phosphatase (INPP1), inositol hexakisphosphate kinase 1 (IP6K1), and inositol-3-phosphate synthase (ISYNA) genes were significantly down regulated at all time periods compared to baseline levels. Inositol hexakisphosphate kinase 2 (IP6K2), inositol hexakisphosphate kinase 3 (IP6K3), and multiple inositol-polyphosphate phosphatase 1 (MINPP1) gene expression, however, were up regulated at 6–8 h post-prandial and then returned to the baseline levels (Figures 5A–F). In the feather, the expression of INPP1 and IP6K1 was induced by 8 h post-prandial and remained higher compared to the baseline (Figures 6A,B). The expression of IP6K2, IP6K3, ISYNA, and MINPP1 was down regulated during the first 10 h and then up regulated for the remainder of the post-prandial period (Figures 6C–F).

## Discussion

Phytate is an important anti-nutritive component in poultry feeds, and adding supplementary phytase to the diet has become broadly utilized in the poultry industry (Bedford, 2000). However, there is a paucity of information regarding the kinetics of phytate hydrolysis to myo-Ins, as well as the role of myo-Ins in poultry metabolism. Here, we were able to monitor myo-Ins kinetic variations in both circulation and feather after the addition of phytase to the diet and have shown marked increases 6 h post-prandial. The majority of dietary myo-Ins that is absorbed is likely transferred to the bloodstream (Lewin et al., 1976), making its presence in circulation a viable marker for digestion of phytate in the small intestine. Indeed, it has recently been shown that plasma myo-Ins levels are greatly increased with phytase feeding at both moderate (Schmeisser et al., 2017) and “super-dosing” levels (Coweison et al., 2015) in chicken. However, even with high levels of phytase, not all phytate is hydrolyzed to myo-Ins, as differently phosphorylated inositol phosphates can be found in the chicken digestive tract (Sommerfeld et al., 2018a, b); therefore, myo-Ins absorption cannot be assumed from the amount of phytate in feed alone, indicating the need for a more direct measurement.

In mammals, circulating myo-Ins can reach body tissues, where it has been shown to accumulate as free myo-Ins (Lewin et al., 1976; Sakamoto et al., 1993), but at varying levels, depending on the tissue, with cerebellar and cortical tissue highest in concentration and muscle and liver lowest (Battaglia et al., 1961). To date, ours is the first report of myo-Ins concentrations in any poultry tissue (Gonzalez-Uarquin et al., 2019). Here, we show that feather myo-Ins increased in a manner similar to that in plasma in the initial (first 10 h) post-prandial state, indicating its potential as a non-invasive method to temporally measure and monitor phytate and myo-Ins metabolism in chicken. Feather sampling has several advantages over other methods of monitoring responses in birds. Feather samples are quick and easy to obtain, do not require significant restraint and stress, and allow for observing the same animal over time. Feather sampling is becoming recognized in this respect, as we have recently shown feather to be advantageous for monitoring HSP70 during heat stress in chicken (Greene et al., 2019), and others have used feather to diagnose Marek’s disease (Davidson and Borenshtain, 2002; Angamuthu et al., 2012), and measure corticosterone (Carbajal et al., 2014; Weimer et al., 2018).

Dietary phytase has positive effects beyond those expected from relief of phytate anti-nutrient effects alone. These effects may be attributable to the hydrolysis of phytate to myo-Ins, which likely has numerous metabolic functions that can affect the growth performance of poultry. As the primary functions of myo-Ins are in cellular signaling pathways and as a precursor of other biological compounds, but it can be synthesized by the body, it may be considered a semi-essential nutrient that may be limited under certain physiological conditions (Gonzalez-Uarquin et al., 2019). In humans, myo-Ins has been shown to potentially reduce the symptoms of metabolic syndrome, including insulin resistance and dyslipidemia (Croze and Soulage, 2013), as well as affect other endocrine disorders such as polycystic ovary syndrome (Unfer et al., 2012), all of which relate to its role as an insulin mimetic. As modern chickens are insulin resistant (Stout et al., 1973; Scanes, 2009; Shiraishi et al., 2011), myo-Ins may be both a viable marker and treatment to help mitigate the negative consequences of this problem in both health and production parameters. Indeed, as myo-Ins may prevent ectopic fat deposition in mice (Plows et al., 2017) and deficiency has been associated with increased hepatic triglycerides in rat (Hayashi et al., 1974), it is conceivable that it may also be correlated with fat deposition in poultry. Though this has yet to be explored, Gonzalez-Uarquin et al. (2019) suggest tracking myo-Ins over the lifespan of the bird in order to monitor lipid metabolism.

Intracellular levels of myo-Ins depend on intestinal absorption, cellular synthesis, and excretion (Croze and Soulage, 2013; Dinicola et al., 2017). Intracellular myo-Ins can be formed in two ways: *de novo* synthesis from glucose and hydrolysis of phosphatidylinositol, phosphoinositides, and inositol phosphates (Loewus and Loewus, 1983). In this study, supplementary phytase or myo-Ins increased the concentrations of myo-Ins in circulation and feather, likely decreasing the need for further synthesis. Evidence for down regulation of both the cellular *de novo* and hydrolysis pathways is evident in the initial post-prandial decreases in gene expression of ISYNA and INPP1 in blood and MINPP1 in both blood and feather. Microbial phytases have varying ability to de-phosphorylate phytate, with some yielding to InsP3 (Haefner et al., 2005), and others able to yield InsP1 (Wyss et al., 1999). Interestingly however, avian MINPP1 has phytase hydrolyzing activity 100 fold greater than its mammalian counterpart (Cho et al., 2006), and is able to convert 80% of phytate to InsP1 (Cho et al., 2006).

The inositol hexakisphosphate kinases (IP6K1-3) are flexible in their function. These enzymes generate inositol pyrophosphates from InsP5, but also dephosphorylate InsP6 to InsP5 in the presence of a decreasing ATP/ADP ratio (Wundenberg et al., 2014). In both blood and feather, there was an immediate post-prandial decrease in the gene expression of IP6K1, IP6K2, and IP6K3. This may reflect the positive energy balance associated with food consumption or the reduction in need for inositol pyrophosphates. These enzymes also play roles in insulin sensitivity (Kamimura et al., 2004; Illies et al., 2007) and apoptosis (Thomas and Potter, 2014), which may help explain the physiological effects of myo-Ins.

In summary, this is the first report, to our knowledge, showing kinetic variations of myo-Ins in both circulation and feathers and suggest feather myo-Ins levels as a reliable non-invasive marker to study phytate hydrolysis in chickens. These findings can allow for better understanding of phytase usage and phytate metabolism in poultry health, disease, and productivity.

## Data Availability Statement

All datasets presented in this study are included in the article/supplementary material.

## Ethics Statement

The animal study was reviewed and approved by Institutional Animal Care and Use Committee at the University of Arkansas.

## Author Contributions

SD conceived and designed the study. EG, BM, and SD conducted the experiments, determined gene and protein expression, and analyzed the data. JW and AC provided the HiPhos. SD and EG wrote the manuscript.

## Conflict of Interest

The remaining authors declare that the research was conducted in the absence of any commercial or financial relationships that could be construed as a potential conflict of interest.
